# ZrO_2_ Superhydrophobic Coating with an Excellent Corrosion Resistance and Stable Degradation Performance on Zr-Based Bulk Metallic Glass

**DOI:** 10.3390/ma17010118

**Published:** 2023-12-26

**Authors:** Ranfeng Wei, Rui Zheng, Chaojun Li, Wei Wang, Hao Zhang, Qijing Sun, Jingwang Lv, Guoyang Zhang, Li Liu, Xiangjin Zhao

**Affiliations:** 1 College of Nuclear Equipment and Nuclear Engineering, Yantai University, No. 30 Qingquan Road, Yantai 264005, China; weiranfeng@s.ytu.edu.cn (R.W.); lcj88888@s.ytu.edu.cn (C.L.); sdxcwang@s.ytu.edu.cn (W.W.); sunqijing@ytu.edu.cn (Q.S.); jingwanglv@ytu.edu.cn (J.L.);; 2 School of Environmental and Material Engineering, Yantai University, No. 30 Qingquan Road, Yantai 264005, China; zrchilling@s.ytu.edu.cn (R.Z.); haozhang@s.ytu.edu.cn (H.Z.)

**Keywords:** Zr-based bulk metallic glass, photocatalytic degradation, electrochemical etching, corrosion resistance, stable degradation

## Abstract

Photocatalysis is an energy-saving and high-efficiency green environmental technology. Because of its wide band gap and low light utilization, few studies have been conducted on ZrO_2_ used as a photocatalytic material. In this paper, a corrosion-resistant superhydrophobic ZrO_2_ coating was prepared on the surface of Zr-based bulk metallic glass by electrochemical etching. This coating not only showed a better corrosion resistance and easier collection, but also presented a stable degradation performance when combined with H_2_O_2_; these characteristics are necessary for photocatalysts to survive under harsh environments. This study provides a new direction for designing superhydrophobic surfaces on bulk metallic glass that possess a functional performance.

## 1. Introduction

Zirconium is a type of transition metal, and its related oxides mostly exist in the form of ZrO_2_ [1]. Transition metal oxides have attracted a lot of attention in recent years due to their stability as photocatalytic materials [2]. Unlike other transition metal oxides used as photocatalytic materials, ZrO_2_ has a wider band gap and lower light utilization, resulting in relatively limited research in the field of photocatalysis [3]. However, under the synergistic effect of H_2_O_2_, the generation of free radicals can be increased and the efficiency of photocatalytic degradation can be improved [4,5]. Furthermore, ZrO_2_ is non-toxic and has an excellent stability that can meet the requirements for catalyst application in the environment [6].

Many studies have been carried out on the preparation of ZrO_2_. Chemical methods are the most common method to prepare ZrO_2_ nanoparticles [1]. However, this always leads to the common problems of powder photocatalysts, such as high dispersion and difficulty in recovery [7]. Preparing oxide coatings on alloy substrates through electrochemical etching is an ideal approach to address the aforementioned drawbacks. In order to adapt to the complex and harsh application environment, corrosion resistance is one of the most important properties when selecting etched alloy substrates. Compared with traditional alloys, metallic glass (MG) has a better corrosion resistance due to the absence of grain boundaries. Zr-based bulk metallic glasses (BMGs), due to their unique property profile, such as their superior glass forming ability and excellent corrosion resistance [8,9], are an excellent option for preparing ZrO_2_ corrosion-resistant coatings using electrochemical etching method. Furthermore, combined with surface modification, the electrochemical etching method has good repeatability, and adjusting the process parameters can easily achieve superhydrophobicity of the coating, thereby providing protection to the material [10]. Unlike other studies on improving the corrosion resistance of Zr-based MGs [11,12], the superhydrophobic coating prepared in this work has the function of accelerating the degradation of pollutants.

In this work, a simple and highly repetitive electrochemical etching method was used to prepare superhydrophobic coatings on Zr-based BMG substrates. The prepared superhydrophobic coating not only improved the corrosion resistance of the material surface, but also had a good degradation performance due to the synergistic effect of H_2_O_2_. Because of the superhydrophobicity of the coating, it could effectively protect the composition and structure of the coating and maintain a stable cycling degradation efficiency. Compared with traditional powder catalysts, the functionalized superhydrophobic coating was built on a BMG substrate, making it easier to collect and compensating for the shortcomings of powder photocatalysts, achieving the integration of structure and function.

## 2. Experimental Methods

### 2.1. Preparation of Coating

**Figure 1:**
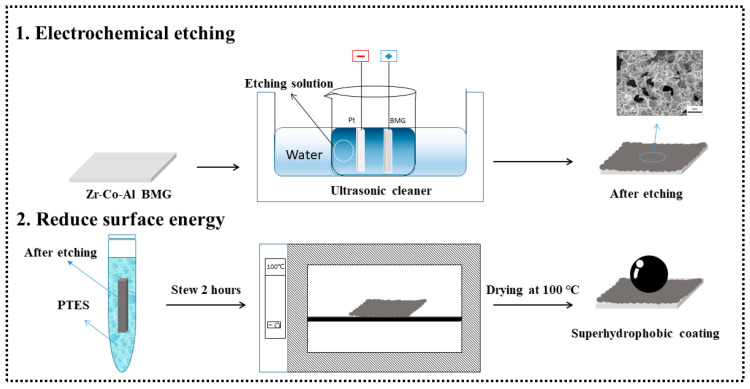
Schematic diagram of the preparation process of the Zr-based BMG superhydrophobic coating.

Zr_56_Al_16_Co_28_ BMGs with a size of 70 mm × 10 mm × 1.5 mm were prepared using the copper mold suction casting method and the purity of Zr, Al, and Co metals used for preparation was better than 99.9 wt%. A diamond cutting machine was used to cut BMG into blocks with dimensions of 10 mm × 10 mm × 1.5 mm, which served as the substrate for preparing superhydrophobic coatings. The original sample was characterized by XRD, which showed only a diffraction hump, indicating that the sample was identified as MG (Figure S1a). Figure 1 shows the process of preparing the superhydrophobic coating on the Zr_56_Al_16_Co_28_ BMG substrate. Firstly, the sample was electrochemically etched. The etching solution was a mixed solution with 0.2 mol/L NaCl and 0.3 mol/L (NH_4_)_2_SO_4_. The square BMG (10 mm × 10 mm) was used as the anode, while the cathode was a pure Pt metal. The entire system was in an ultrasonic environment with a power of 100 W and a frequency of 100 kHz, with the temperature set at 299 K, a distance between the two poles of 10 mm, a constant current of 0.6 A/cm^2^, and an etching time of 14 min. The electrochemical-etched sample was cleaned in deionized water and anhydrous ethanol, and then dried (373 K) in an oven (H1310, Jinghong, Shanghai, China). Secondly, after cooling to room temperature, the sample was soaked in 1.0 wt% 1-H, 1-H, 2H, and 2H-Perfluorooctyltriethoxysilane (PTES) for over 2 h, and then dried again at 373 K to reduce the surface energy.

### 2.2. Characterizations

The contact angle (CA) and sliding angle (SA) of the coating were measured using a contact angle measuring instrument (JC2000C1, Zhongchen, Shanghai, China). The corrosion resistance was evaluated using an electrochemical workstation (CHI660E, Chenhua, Shanghai, China). The degradation performance test was conducted using methyl orange (MO) as the pollutant model at a stable temperature of 303 K. A 300 W Xe lamp (CEL-HXF300-T3, Beijing China Education Au-light Co., Ltd., Beijing, China) was used as the light source (350 nm ≤ λ ≤ 780 nm), 15 cm away from the solution, and the absorbance of the solution was measured using a UV spectrophotometer (UV-5500, Bio-Equip, Shanghai, China). The microstructure of the coating was characterized by scanning electron microscope (SEM, JSM-7610 F, JEOL, Tokyo, Japan). The chemical composition was analyzed by energy dispersive spectroscopy (EDS; JSM-7610 F, Japan), X-ray photoelectron spectroscopy (XPS; Thermo escalab 250, American, and the scanning rate was 0.05 eV/s), and X-ray diffraction (XRD; MiniFlex600, Rigaku, Tokyo, Japan) analysis.

## 3. Results and Discussion

**Figure 2:**
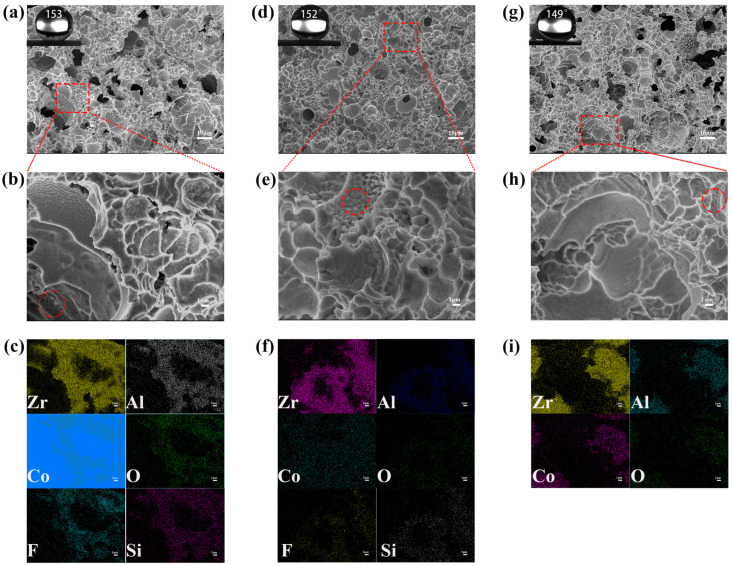
SEM images of the surface and EDS elemental mapping images: (**a**–**c**) original superhydrophobic coating, (**d**–**f**) coating after soaking for 10 days, and (**g**–**i**) coating after cyclic degradation test.

The water droplets were dropped onto the original sample (BMG without any treatment) and they adhered to the surface in a hemispherical shape with CAs of ~80°, indicating that the surface of the original sample was hydrophilic (Figure S2a). The CAs of the coating prepared on BMGs reached ~153° and the SAs were less than 5°, indicating that the coating was superhydrophobic [13] (Figure S2b). The morphology characterization of the superhydrophobic coatings by scanning electron microscopy showed that the BMG coating after electrochemical etching was composed of polygonal protrusions and pits, with micro/nano roughness (Figure 2a), which were crucial for achieving superhydrophobic properties on the material surface [14]. After zooming in on the coating, it was found that the inner walls of the pits were densely packed with small particles (Figure 2b). The XRD pattern for the original superhydrophobic coating only showed a scattering hump (Figure S1b). According to the EDS spectroscopy, in addition to Zr, Al, and Co elements in the original alloy system, O, F, and Si were also detected (Figure S3a). The O element was caused by metal oxidation during the electrochemical etching, while the appearance of F and Si was due to the attachment of the modification solution components to the coating. Zr, Al, Co, and O were detected in the same distribution state, indicating that the surface was uniformly corroded, and F and Si also had the same distribution state, indicating that the modified solution components adhered uniformly to the surface structure, reducing the surface energy of the rough surface (Figure 2c). The synergistic effect of the rough surfaces and low surface energy led to the superhydrophobicity of the surface [15].

**Figure 3:**
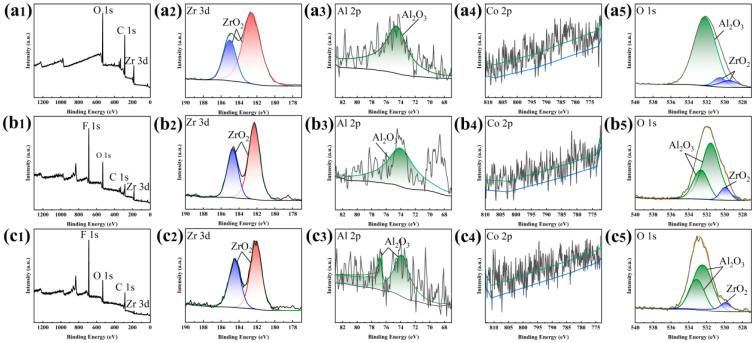
(**a1**–**a5**) XPS spectra of the sample surface after electrochemical etching; (**b1**–**b5**) XPS spectra of the sample surface after soaking in 3.5 wt% NaCl for 10 days; (**c1**–**c5**) XPS spectra of the sample surface after cyclic degradation test.

To further investigate the surface composition of the samples, the unmodified anode samples were characterized by XPS; the analysis used the AVANTAGE 5.99 software and the database was affiliated with the software. Figure 3(a1) shows the etched anode surface was mainly composed of Zr, C, and O. Further fine sweep spectroscopy revealed two peaks in the Zr 3d spectrum, 182.52 eV and 185.04 eV, which corresponded to the binding energy of ZrO_2_ (Figure 3(a2)). A peak in the Al 2p spectrum was displayed at 74.57 eV, which corresponded to the binding energy of Al_2_O_3_ (Figure 3(a3)). However, the resolution of the fine scanning spectra of Co was low, which made it difficult to analyze the specific positions of the peaks (Figure 3(a4)). The peaks in the O1s spectrum also corresponded to ZrO_2_ and Al_2_O_3_ (Figure 3(a5)).

**Figure 4:**
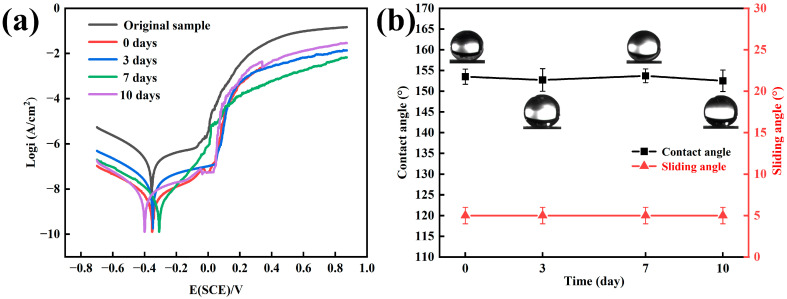
(**a**) Polarization curves of the samples soaked in 3.5 wt% NaCl over a period of various days; (**b**) CA changes of the samples soaked in 3.5 wt% NaCl over a period of various days.

Figure 4a shows the results of the potentiodynamic polarization test of the samples in a 3.5 wt% NaCl solution soaked for 0 days (original superhydrophobic sample) to 10 days (the working electrode was the sample to be tested, the reference electrode was the calomel electrode, the auxiliary electrode was Pt, and the scanning speed was 0.01 V/s). The corrosion inhibition rate can be calculated using the following formula [16]:

$$\eta =\frac{{R_{P}}^{inh}-{R_{P}}^{blank}}{{R_{P}}^{inh}}$$

*η* is the corrosion inhibition efficiency, ${R_{P}}^{inh}$ is the polarization resistance of the superhydrophobic coating, and ${R_{P}}^{blank}$ is the polarization resistance of the original sample. Compared with the original sample, the inhibition efficiency of the superhydrophobic coating was 99.00%, indicating that the successful preparation of the superhydrophobic coating improved the corrosion resistance of the BMG surface. Compared with the method of adjusting the Zr-based BMG system [17,18], the superhydrophobic coating prepared in this work improved the corrosion resistance of the BMG more effectively.

Figure 4b shows that the soaking time was extended 10 days, and there was no significant change in the contact angle; the water droplets could smoothly roll on the coating, indicating that the coating maintained a positive hydrophobicity. Compared with the original sample, the superhydrophobic sample soaked in 3.5 wt% NaCl solution for different days still maintained a lower current, indicating a better corrosion resistance than the original sample (Figure 4a).

**Figure 5:**
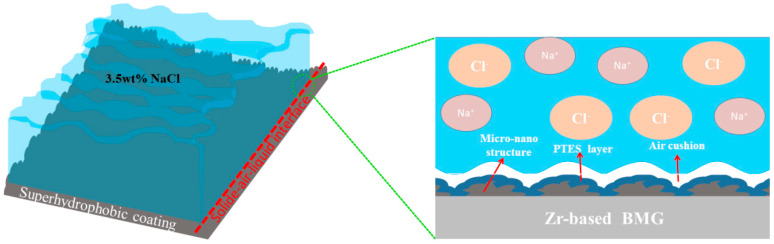
Solid−air−liquid interface in the 3.5 wt% NaCl solution.

According to the morphology and chemical composition of the coating after 10 days, there were almost no changes in the surface structure (Figure 2d,e), and the F and Si elements that were detected through EDS (Figure S3b) were still uniformly distributed (Figure 2f). This indicates that there was no significant change in the coating structure and the modification solution components still adhered well to the structure. Similar to the original superhydrophobic coating, the samples remained amorphous after soaking in a 3.5 wt% NaCl solution for 10 days, as seen from the XRD trace (Figure S1c). The XPS results show that the surface was mainly composed of F, Zr, C, and O (Figure 3(b1)). The resolution of the Co fine scanning spectrum was still very low, as shown by the fine scanning spectrum (Figure 3(b4)). And Zr 3d still existed in the form of ZrO_2_, corresponding to the two peaks at 182.24 eV and 184.71 eV **(**Figure 3(b2)). And one peak was displayed in the Al 2p spectrum at 73.99 eV, which corresponded to the binding energy of Al_2_O_3_ (Figure 3(b3)). And O 1s also corresponded to ZrO_2_ and Al_2_O_3_, with no other oxides (Figure 3(b5)). The above results indicate that the coating was not significantly corroded; the efficiency in subsequent degradation experiments was guaranteed precisely because of the stability of the coating. As shown in Figure 5, when the sample was immersed in a 3.5 wt% NaCl solution, the micro/nano structure on the coating surface captured air to form an air cushion, repelling the solution and forming a solid−air−liquid interface [19], reducing the solid−liquid contact area and providing good protection for the coating. This was a supporting condition for maintaining a stable hydrophobic angle and corrosion resistance.

**Figure 6:**
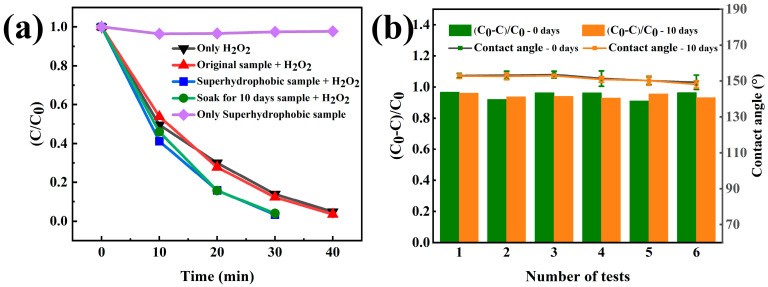
(**a**) Photocatalytic activity under different conditions and (**b**) histogram of cyclic degradation efficiency and line chart of CAs change.

In order to compare the degradation rate of the MO solution (the volume of MO was 10 mL), the C/C_0_ Line chart diagram of the degradation rate is shown in Figure 6a. The curves, which were used to calculate the degradation rate, are shown in Figure S4. C_0_ is the initial concentration of MO and C is the concentration of MO at reaction time t. As shown in Figure 6a, the degradation percentage of the MO solution with the superhydrophobic sample (purple curve) did not change significantly with time. The solution completely degraded after 40 min with 0.1 mL H_2_O_2_ (black curve). It took a similar time to completely degrade when the original sample and 0.1 mL H_2_O_2_ were added (red curve). However, when the superhydrophobic sample and 0.1 mL H_2_O_2_ were added, the solution completely degraded after 30 min (blue curve). This indicates that the combined effect of H_2_O_2_ and superhydrophobic coatings could improve the degradation rate significantly. The degradation performance of the sample soaked for 10 days in a 3.5 wt% NaCl solution was also tested, and its degradation performance was similar to that of the initial superhydrophobic sample (green curve). This indicates that the sample still had an excellent degradation performance after the soaking corrosion. Based on the above experimental results and component analysis, the reaction formula for accelerating the degradation process can be inferred as follows [3,20,21]:

ZrO_2_ + hν → e^−^ + h^+^

h^+^ + H_2_O → •OH +H^+^

e^−^ + O_2_→•O_2_^−^

H_2_O_2_ + e^−^ → •OH + OH^−^

•O_2_^−^ + 2H^+^ + 2e^−^ → •OH + OH^−^

H_2_O_2_ + hν → •OH

•OH + MO → CO_2_ + H_2_O

In order to evaluate the degradation stability of the coating, cyclic degradation experiments were conducted. The results showed that after six cycles of degradation, the hydrophobic angle showed a downward trend, but the degradation efficiency remained stable. The microstructure and chemical composition of the coating after cyclic degradation were characterized. The microstructure of the original superhydrophobic coating after six degradation cycles showed no significant difference compared with the original superhydrophobic coating (Figure 2g,h). No new diffraction humps were detected using XRD, and the amorphous phases still existed (Figure S1d). However, no obvious peaks of F and Si were detected through EDS (Figure S3c), while the remaining Zr, Al, Co, and O were uniformly distributed, indicating that the decrease in the hydrophobicity angle in the cyclic degradation experiment was due to the loss of modification liquid components (Figure 2i). XPS was used for a more precise analysis of the coating components. The results in Figure 3c show the same oxide composition as the original coating, with no new oxide generation (Figure 3a). Peaks were apparent in the Zr 3d spectrum and Al 2p spectrum at 182.06 eV, 184.48 eV, 73.78 eV and 76.81 eV, which correspond to the binding energy of ZrO_2_ and Al_2_O_3_, and F element was detected (Figure 3(c2,c3)). Figure 3(c4) shows that the relevant Co material remained unchanged, but the O1s spectrum still corresponded to the oxides of ZrO_2_ and Al_2_O_3_ (Figure 3(c5)). After cyclic degradation, the sample with reduced hydrophobic angle was further modified, and the contact angle returned to the superhydrophobic level. The results validate the previous speculation, indicating that the decrease in angle after cyclic degradation was caused by a decrease in the content of modified components on the surface structure.

## 4. Conclusions

In this paper, a superhydrophobic coating was prepared on the surface of Zr-based MGs by electrochemical etching. The hydrophobicity, corrosion resistance, and degradation performance of superhydrophobic coatings were tested. The following conclusions can be drawn.

(1) The prepared superhydrophobic coating on the substrate improved the degradation efficiency of the pollutants and compensated for the lack of dispersion of powder catalysts.

(2) The superhydrophobic coating significantly improved the corrosion resistance of the surface, maintaining an excellent hydrophobicity even after soaking in 3.5 wt% NaCl for 10 days, and possessing the same degradation efficiency as the original superhydrophobic coating.

(3) The cyclic degradation efficiency of the coating was stable. It could achieve six cycles of degradation while ensuring a good hydrophobicity.

## Data Availability

Data are contained within the article.

## Supplementary Materials

The following supporting information can be downloaded at: https://www.mdpi.com/article/10.3390/ma17010118/s1, Figure S1: The XRD pattern of sample surface: (a) Original metal glass, (b) Original superhydrophobic coating, (c) Coating after soaking for 10 days, (d) Coating after cyclic degradation test. Figure S2: (a) Water droplets on the surface of the original sample, (b) Water droplets on the surface of the superhydrophobic sample. Figure S3: The EDS spectrum of the surface: (a) Original superhydrophobic coating, (b) Coating after soaking for 10 days, (c) Coating after cyclic degradation test. Figure S4: Time dependent UV spectra of MO solution under different conditions: (a) only superhydrophobic sample, (b) only H_2_O_2_, (c) original sample and H_2_O_2_, (d) original superhydrophobic sample and H_2_O_2_, (e) superhydrophobic sample after soaking for 10 days and H_2_O_2_.
