# Supplementary material for: ZrO2 Superhydrophobic Coating with an Excellent Corrosion Resistance and Stable Degradation Performance on Zr-Based Bulk Metallic Glass
Source: Materials (Basel). 2023 Dec 26;17(1):118. doi: 10.3390/ma17010118 (PMC10779633; doi:10.3390/ma17010118)
Supplement: Supplementary file 1 [file materials-17-00118-s001.zip › materials-2766449-supplementary.pdf]

ZrO_2 superhydrophobic coating with excellent corrosion resistance and stable degradation performance on Zr-based bulk metallic glass

Ranfeng Wei[a], Rui Zheng[b], Chaojun Li[a], Wei Wang[a], Hao Zhang[b], Qijing Sun[a], Jingwang Lv[a], Guoyang Zhang[a], Li Liu[b,*], Xiangjin Zhao[a,*]

[a] College of Nuclear Equipment and Nuclear Engineering, Yantai University, No. 30 Qingquan Road, Shandong, 264005, China

[b] School of Environmental and Material Engineering, Yantai University, No. 30 Qingquan Road, Shandong, 264005, China

*Corresponding author.

E-mail address: 15053532471@163.com (L. Liu); zl2915@ytu.edu.cn (X.J. Zhao).

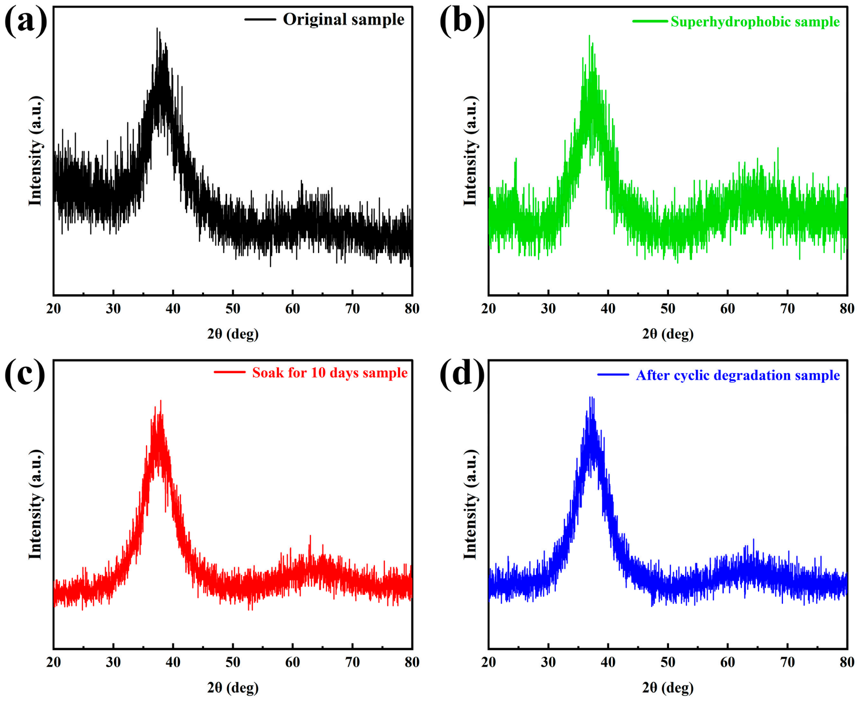

Figure S1 The XRD pattern of sample surface: (a) Original metal glass, (b) Original superhydrophobic coating, (c) Coating after soaking for 10 days, (d) Coating after cyclic degradation test

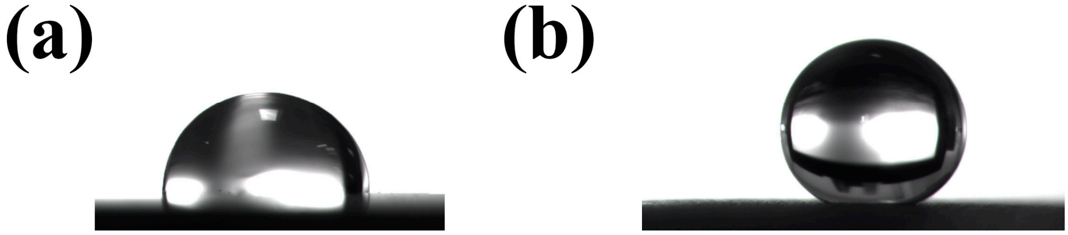

Figure S2 (a) Water droplets on the surface of the original sample, (b) Water droplets on the surface of the superhydrophobic sample.

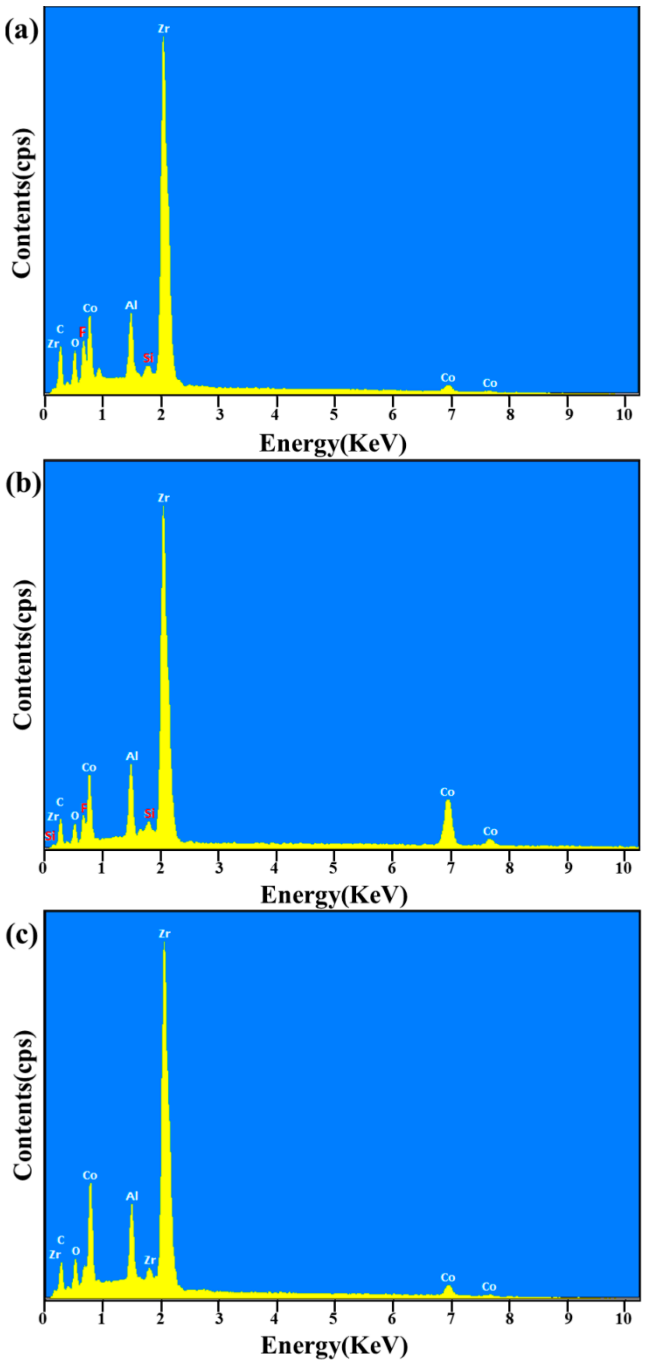

Figure S3 The EDS spectrum of the surface: (a) Original superhydrophobic coating, (b) Coating after soaking for 10 days, (c) Coating after cyclic degradation test.

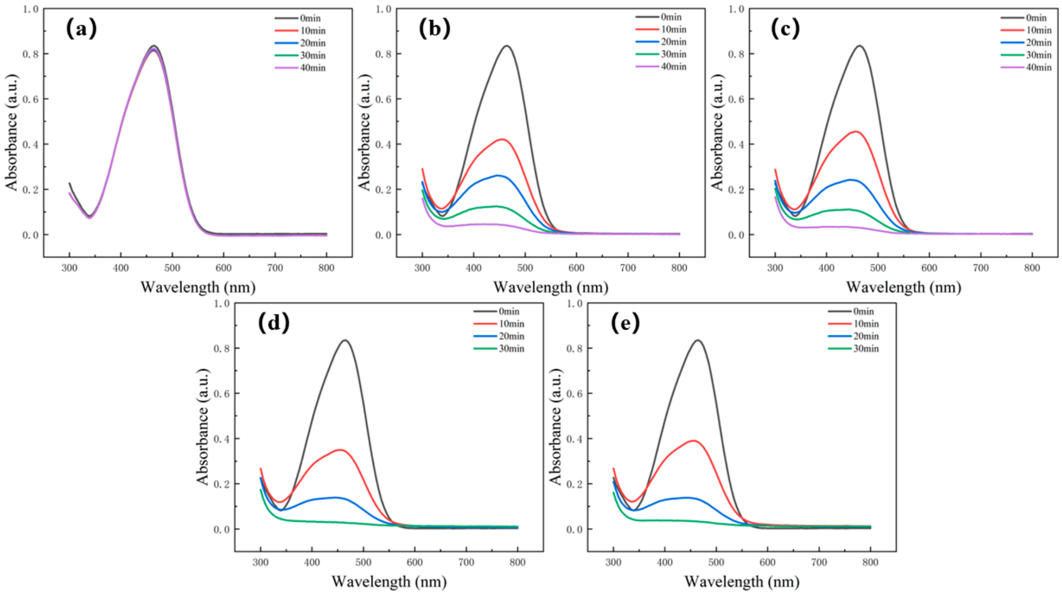

Figure S4 Time dependent UV spectra of MO solution under different conditions：
(a) only superhydrophobic sample, (b) only H_2O_2, (c) original sample and H_2O_2, (d) original superhydrophobic sample and H_2O_2, (e) superhydrophobic sample after soaking for 10 days and H_2O_2.
